# Investigating the Relationship Between Hypertension and Obesity in Schoolchildren From Lower-Middle Socioeconomic Strata in Urban Mumbai

**DOI:** 10.7759/cureus.55203

**Published:** 2024-02-29

**Authors:** Ashish Goel, Paula Goel

**Affiliations:** 1 Department of Cardiology, Fayth Clinic Medical Institute, Mumbai, IND; 2 Department of Pediatrics, Fayth Clinic Medical Institute, Mumbai, IND

**Keywords:** pre-hypertension, urban india, bmi, underweight, overweight, obesity, hypertension

## Abstract

Background

In light of escalating rates of childhood obesity, understanding the gender-specific correlation between body mass index (BMI) and hypertension has become crucial for effective public health interventions. This study investigates the interplay between BMI and hypertension among school-aged children, with a particular emphasis on gender stratification to identify distinct trends.

Methodology

A cross-sectional study was conducted with a diverse sample of 702 schoolchildren aged 5-16 years from a lower-middle-income school in urban Mumbai. This cohort consisted of 491 boys and 211 girls within the gender subset. BMI was calculated using height and weight measurements, while blood pressure readings determined hypertension prevalence. The children were categorized based on the Indian Academy of Pediatrics (IAP) growth chart BMI calculations and blood pressure percentiles. SPSS Statistics version 23 (IBM Corp., Armonk, NY, USA) was used for data analysis. Data were analyzed using the chi-square test, with p-values <0.05 deemed significant.

Results

The overall prevalence of overweight was 16.52%, with 15.89% in boys and 18.10% in girls, revealing no significant gender difference (p = 0.487). In terms of obesity, the overall prevalence was 10.83%, with 10.99% in boys and 10.34% in girls, revealing no significant gender difference (p = 0.823). The prevalence of pre-hypertension was 7%, exhibiting a significantly higher prevalence in high BMI males (overweight and obese) versus non-high BMI males (normal and underweight) (p < 0.001); however, no such difference was observed in females (p = 0.289). The prevalence of hypertension was 15.95% with a significantly higher prevalence in high BMI males (overweight and obese) versus non-high BMI males (normal and underweight) (p < 0.001) and high BMI females (overweight and obese) versus non-high BMI females (normal and underweight) (p < 0.001). Hypertension was significantly higher in children with high BMI (overweight and obese) compared to their non-high BMI (normal and underweight) counterparts.

Conclusions

In lower-middle socioeconomic strata schoolchildren in urban Mumbai, the prevalence of obesity and hypertension was alarmingly high, attributed to shifting lifestyles and unhealthy dietary habits. Hypertension rates were notably elevated among overweight and obese individuals compared to normal and underweight individuals. More than a third of both boys and girls with obesity were diagnosed with hypertension, emphasizing a concerning surge in hypertension cases among children. Prioritizing age-specific blood pressure assessments can facilitate early identification and timely interventions.

## Introduction

With the escalating prevalence of childhood obesity, understanding the gender-specific correlation between body mass index (BMI) and hypertension has become crucial for devising effective public health interventions. Screening for hypertension in children and adolescents is gaining heightened significance [1] due to the increasing challenges posed by elevated BMI and obesity, leading to a spectrum of health issues [2].

Both globally and within India, the evaluation of hypertension in children and adolescents, along with its correlation with obesity, has been an area of study for over four decades [3-5]. Recognizing the enduring relevance of this research, the assessment of hypertension in the young population has become increasingly vital. Incorporating blood pressure screening as a mandatory component of the annual health check and integrating it into school health screening processes can effectively identify individuals with hypertension and high BMI using validated screening methods [6]. Notably, blood pressure changes with the age and sex of the child, as well as trends indicating a rise in blood pressure [7] with advancing age, emphasize the need for accurate measurement standards.

Our study used the blood pressure screening norms outlined in the Fourth Report on the Diagnosis, Evaluation, and Treatment of High Blood Pressure in Children and Adolescents [8] which have been widely adopted, including in India, the United States, Africa, and Asia. Despite some modifications to these guidelines in practice [9], they continue to be influential across various regions [10-14].

This study endeavors to explore and analyze the intricate relationship between high BMI and hypertension in school-aged children, with a focus on gender-specific patterns. The objectives include examining the distribution of BMI categories among boys and girls in the school-aged population, assessing the prevalence of hypertension among both genders across different BMI categories, and scrutinizing the correlation between BMI and hypertension, particularly exploring gender differences.

## Materials and methods

Study design

In this cross-sectional study, a diverse sample of 702 school-aged children was enrolled, with particular attention given to gender stratification. The cohort comprised 491 boys and 211 girls. BMI calculation involved height and weight measurements, while blood pressure readings were taken to assess the prevalence of hypertension.

Data collection

Standardized protocols were employed to collect demographic information, BMI measurements, and blood pressure readings. The study prioritized ethical considerations and participant confidentiality throughout the entire process.

Data collection protocols

Demographic Information

Informed consent forms were distributed to parents or legal guardians before data collection, outlining the study goals and procedures. Participation was restricted to children with signed consent forms. A confidential questionnaire collected demographic information such as age, gender, ethnicity, and socioeconomic status, providing context for understanding potential disparities in the relationship between BMI and hypertension.

Height and Weight Measurements (BMI)

Trained healthcare professionals or research assistants conducted height and weight measurements using standardized equipment in private settings to ensure participant comfort and confidentiality. BMI was calculated using the following standard formula: BMI = weight (kg)/square of height in meters, and children were categorized based on the Indian Academy of Pediatrics growth chart. Measurements were recorded on individual data forms, and participants were assigned unique identifiers to maintain anonymity.

Blood Pressure Readings

Blood pressure readings were obtained using calibrated sphygmomanometers with appropriate cuff size as per age. Measurements were performed in a quiet and comfortable environment to minimize potential stressors. Three separate readings were obtained for participants with high blood pressure for age, with a brief rest period between measurements to reduce the potential for inaccurate readings due to stress or discomfort. An average of the three readings was used to determine the participant’s blood pressure status. Pre-hypertension in children was defined as average systolic blood pressure (SBP) or diastolic blood pressure (DBP) levels greater than or equal to the 90th percentile but less than the 95th percentile according to specific blood pressure guidelines chart based on age, sex, and height. Hypertension was defined as an average SBP and/or DBP greater than or equal to the 95th percentile for sex, age, and height on three or more occasions.

Ethical considerations

This study adhered to the ethical guidelines outlined by institutional review boards and research standards for human subjects. Informed consent and assent were obtained, confidentiality was strictly maintained, and potential risks were minimized with appropriate support. Personal information was kept separate from research data, with identifiers replaced by unique codes. Strict data security measures were implemented, and personnel underwent training to uphold participant confidentiality. By implementing these robust data collection protocols, the study aimed to ensure research integrity, prioritize participant well-being, and generate reliable and ethically obtained data for analysis.

Data analysis

The collected data were analyzed using SPSS Software version 23 (IBM Corp., Armonk, NY, USA). The chi-square test was employed for data analysis. P-values <0.05 were considered to be statistically significant.

## Results

A total of 702 children participated in the study, with 491 males and 211 females. As shown in Table 1, 16.52% of children were overweight, and 10.83% were obese. No significant differences were observed between boys and girls.

**Table 1:** Distribution of body mass index (BMI). NS: non-significant

BMI category	Total children	Males	Females	P-value
Underweight/Normal	510 (72.65%)	359	151	0.672 (NS)
Overweight	116 (16.52%)	78	38	0.487 (NS)
Obese	76 (10.83%)	54	22	0.823 (NS)

The overall prevalence of pre-hypertension was 7% (49 of 702 children) (Table 2). There was a significantly higher prevalence of pre-hypertension in high BMI (overweight/obese) males compared to non-high BMI (underweight/normal) males (p < 0.001), but this difference was not significant in high BMI (overweight/obese) females compared to non-high BMI (underweight/normal) females (p = 0.289).

**Table 2:** Prevalence of pre-hypertension. NS: non-significant

Pre-hypertension category	Total children with pre-hypertension (n = 49)	Males	Females	P-value (Males)	P-value (Females)
Underweight/Normal with pre-hypertension	19/510 (3.72%)	9/359	10/151	<0.001 (significant)	0.289 (NS)
Overweight with pre-hypertension	19/116 (16.38%)	14/78	5/38		
Obese with pre-hypertension	11/76 (14.47%)	8/54	3/22		

The overall prevalence of hypertension was 15.95% (Table 3). In both males and females, there was a significantly higher prevalence of hypertension in high BMI (overweight/obese) children compared to non-high BMI (underweight/normal) children (p < 0.001).

**Table 3:** Prevalence of hypertension.

Hypertension category	Total children with hypertension (n = 112)	Males	Females	P-value (Males)	P-value (Females)
Underweight/Normal with hypertension	54/510 (10.59%)	46/359	8/151	<0.001 (significant)	<0.001 (significant)
Overweight with hypertension	29/116 (25%)	19/78	10/38		
Obese with hypertension	29/76 (38.16%)	19/54	10/22		

**Figure 1:**
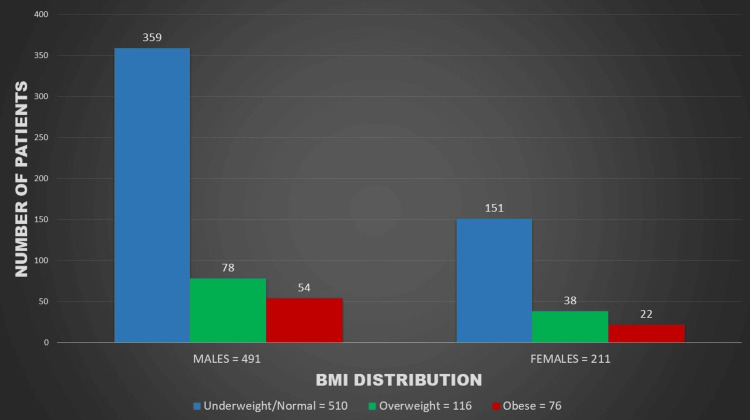
Gender distribution of BMI among study participants. BMI: body mass index

**Figure 2:**
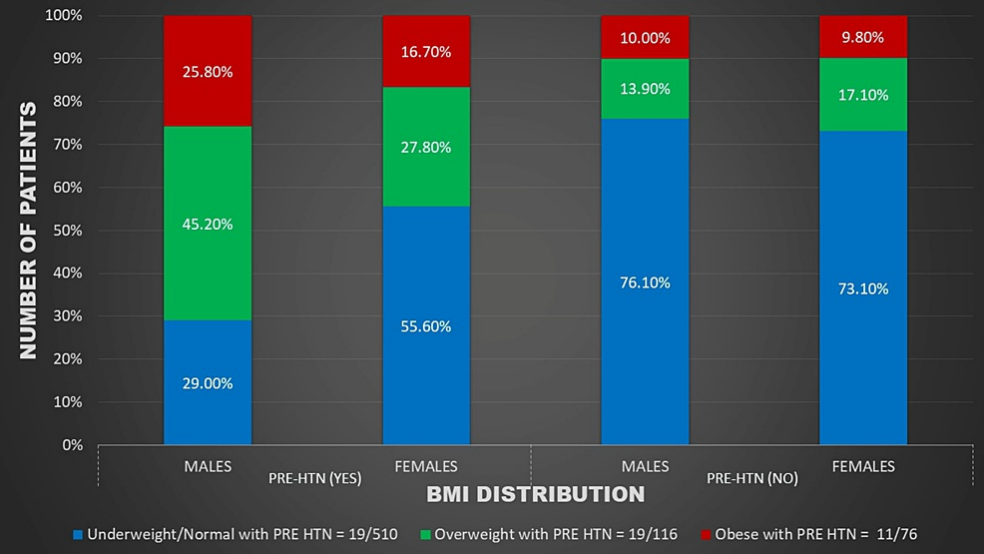
Gender distribution of BMI and pre-hypertension among study participants. BMI: body mass index; PRE HTN: pre-hypertension

**Figure 3:**
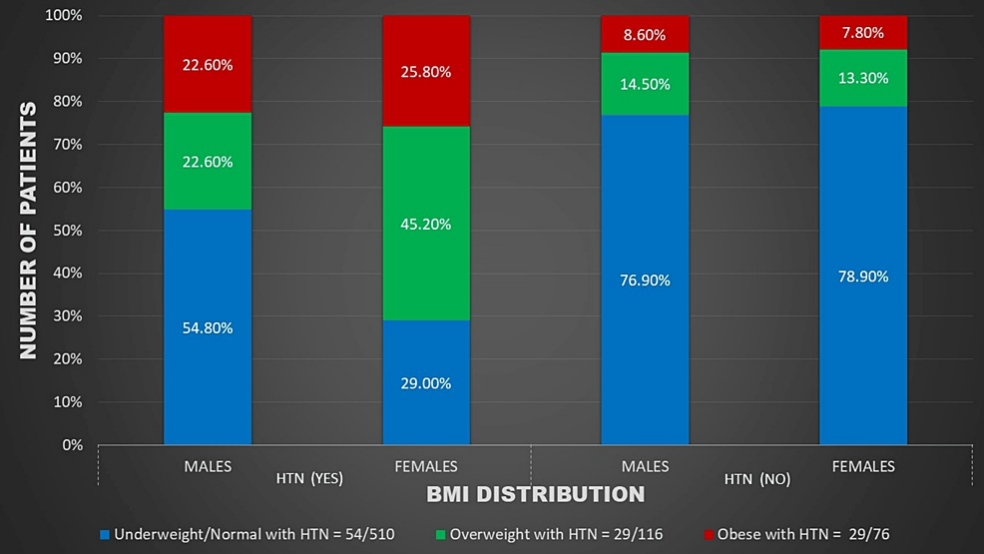
Gender distribution of BMI and hypertension among study participants. BMI: body mass index; HTN: hypertension

**Figure 4:**
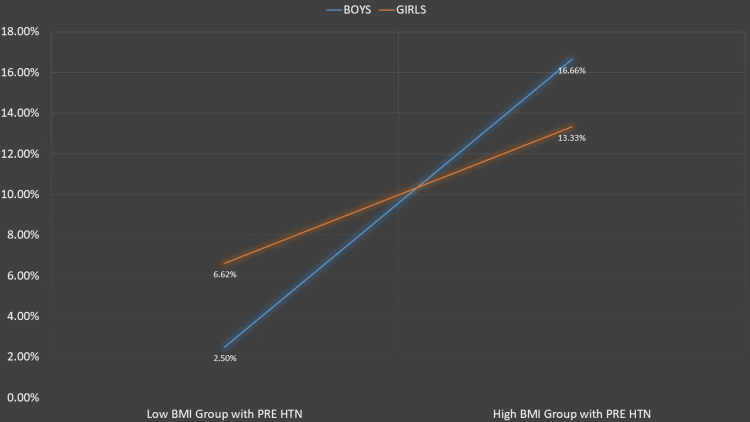
Prevalence of pre-hypertension across BMI groups. Low BMI group: underweight/normal BMI status. High BMI group: overweight/obese BMI status. As shown in Table 2, significant differences in the prevalence of pre-hypertension were observed in males but not in females. BMI: body mass index; PRE HTN: pre-hypertension

**Figure 5:**
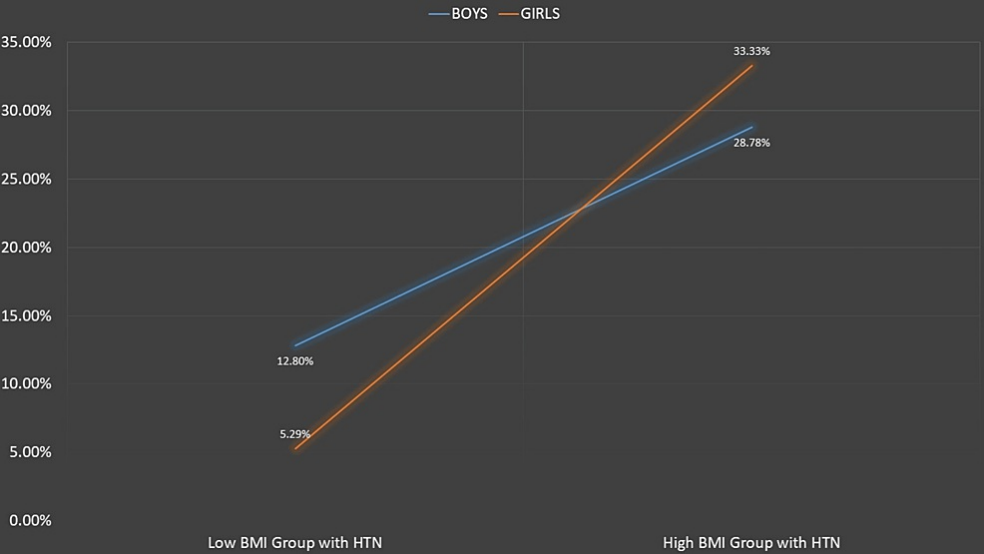
Prevalence of hypertension across BMI groups. Low BMI group: underweight/normal BMI status. High BMI group: overweight/obese BMI status. As shown in Table 3, significant differences in the prevalence of hypertension were observed in both males and females. BMI: body mass index; HTN: hypertension

Figures 1-5 represent the relevant trends and distributions observed in the study.

## Discussion

The escalating prevalence of hypertension and obesity among children is attributed to evolving lifestyles and dietary patterns. This investigation focused on a cohort of students aged 5-16 in an urban Mumbai school, representing the lower-middle-income socioeconomic strata. Adopting the guidelines from the Fourth Report on the Diagnosis, Evaluation, and Treatment of High Blood Pressure in Children and Adolescents [8], we have annually monitored these parameters since 2014. In this study, the prevalence rates of hypertension and pre-hypertension in 2023 have been reported. Utilizing age and sex-specific guidelines is crucial to avoid underdiagnosing high blood pressure in children.

Various Indian studies have explored the prevalence of hypertension and obesity in schoolchildren, examining their interrelation. In our study, the prevalence of high BMI (overweight and obese) exceeded 25% among all children, with no significant gender disparity (Table 1, Figure 1). This finding is particularly noteworthy in the context of children from lower socioeconomic strata, emphasizing the pervasive nature of increasing weight concerns in this demographic.

Our study identified a 7% prevalence of pre-hypertension and an approximately 16% prevalence of hypertension (Tables 2, 3). Comparatively, a study from north India [15] reported a 22.5% prevalence of hypertension among 10-19-year-olds. A meta-analysis encompassing 64 studies [16] reported a pooled prevalence of 7% for hypertension, 4% for sustained hypertension, and 10% for pre-hypertension, with an upward rising trend observed post-2005. Urban children exhibited a higher hypertension prevalence than their rural counterparts, and children with obesity displayed a significantly elevated risk (29%) compared to their normal-weight peers (7%).

Vasudevan et al. [17] in 2022 reported a hypertension prevalence of 35.1% in 10 to 12-year-olds and 25.1% in those aged 13 or older, with both age groups demonstrating increased risk with overweight and obesity.

A study in rural India [18] encompassing 878 children from Tamil Nadu revealed a 5.58% prevalence of hypertension, with a 15.09% prevalence in obese children and 1.35% in overweight children. Similarly, Mohan et al. [19] found a positive association between hypertension and overweight/obesity, reporting a prevalence of 5.7% and 8.4% for sustained hypertension in rural and urban areas, respectively.

Our study reaffirms a robust association between hypertension and weight. The prevalence of pre-hypertension was 16.38% in overweight and 14.47% in obese children, while the prevalence of hypertension was 25% in overweight and 38.16% in obese children. Notably, pre-hypertension was more prevalent in overweight children, particularly males, indicating a trajectory toward a higher prevalence of sustained hypertension as they progress to obesity (Figures 2, 3).

Statistical analysis underscored a highly significant difference in the prevalence of hypertension between overweight/obese children and those who were underweight/normal. The rising prevalence of overweight and obesity in children and adolescents demands increased attention globally and in India, particularly considering the heightened risk of early-onset medical conditions such as hypertension, diabetes, dyslipidemia, and ischemic heart disease in later adulthood, even within lower socioeconomic groups.

Contrary to worldwide trends suggesting higher obesity rates in boys [20], our study found an almost equal distribution of high BMI in both boys and girls. The surge in obesity is linked to changing lifestyles characterized by inadequate physical activity and diets rich in high-fat and high-caloric foods, constituting a significant contributor to this growing health challenge. Addressing these factors is imperative for effective intervention and prevention strategies.

Limitations

Despite adhering to standardized protocols for blood pressure measurements, we acknowledge the inherent variability in readings, especially in a school setting. Factors such as temporary stress or anxiety during measurement can influence results. Although multiple measurements were taken to minimize this, some level of variability may exist. Additionally, while the study considered lower-middle-income socioeconomic status, it did not extensively delve into the nuanced socioeconomic factors that may contribute to obesity and hypertension. A more comprehensive examination of family income, parental education, dietary habits, and physical activity levels could provide a richer understanding of the observed associations.

## Conclusions

Schoolchildren from the lower-middle socioeconomic strata exhibit a significant prevalence of obesity and hypertension, primarily attributed to changing lifestyles and inadequate dietary practices. Notably, hypertension rates are markedly higher in overweight and obese children compared to normal and underweight children, with more than one-third of both boys and girls in the obese category being diagnosed with hypertension. The increasing occurrence of obesity and hypertension in children belonging to lower-middle socioeconomic backgrounds raises significant concerns. Recognizing this growing problem is crucial for devising interventions informed by these observations, as well as for formulating targeted and effective public health strategies.
